# Analysis of phytosterols encapsulated in pegylated liposomes

**DOI:** 10.1038/s41598-025-12722-z

**Published:** 2025-08-13

**Authors:** Joanna Igielska-Kalwat, Magdalena Rudzińska, Anna Grygier, Dominik Kmiecik, Katarzyna Cieślik-Boczula, Krzysztof Dwiecki, Wojciech Smułek

**Affiliations:** 1 Faculty of Food Science and Nutrition, Poznań University of Life Sciences, Wojska Polskiego 28, Poznań, 60-637 Poland; 2 Faculty of Chemistry, University of Wrocław, F. Joliot-Curie 14, Wrocław, 50-383 Poland; 3 Institute of Chemical Technology and Engineering, Poznan University of Technology, Berdychowo 4, Poznań, 60-965 Poland; 4 Polskiego 28, Poznań, 60- 637 Poland

**Keywords:** PEGylated liposomes, Stigmasterol, Fatty acids, Oxidation, Stability, Plant sciences, Health care, Chemistry

## Abstract

Dipalmitoylphosphatidylcholine (DPPC) lipids were encapsulated in PEGylated liposomes with free stigmasterol (ST), stigmasterol myristate (ME), and stigmasterol oleate (OE). Their quality was assessed using TEM, zeta potential, and hydrodynamic diameter measurements. The liposomes were heated to 60 °C and 180 °C. The degradation of stigmasterol and fatty acids was considered, as was derivative formation. The results show that the liposomes fulfilled their intended function. The ST liposomes were smallest, while the ME liposomes were similar in size to the OE liposomes. The degree of degradation of the compounds encapsulated in the liposomes depended on their structure. After heating the samples to 60 °C, the extent of stigmasterol degradation ranged from 3.5% in ST to 4.3% in ME and 6.5% in OE. After heating to 180 °C, the lowest level of stigmasterol degradation was observed for OE (7.3%), while degradation in ST and ME reached 13.4% and 10.1%, respectively. The high level of oxyphytosterols in all samples heated to 180 °C raised concerns. The oxyphytosterol (SOP) content of the liposomes heated to 60 °C ranged from 23.2 mg/g in those with free stigmasterol to 6.3 mg/g and 6.4 mg/g in the liposomes with stigmasterol myristate and stigmasterol oleate, respectively. After heating to 180 °C, the total SOP content was significantly higher, ranging from 88.7 mg/g for OE to 7.4 and 29.6 mg/g for ME and ST, respectively.

## Introduction

Long-term research on liposomes has confirmed their effectiveness as delivery systems for drugs, cosmetics, and many biologically active substances^1–3^. This is primarily due to the similarity of the lipid bilayer structures of the vesicles to the structure of natural biological membranes. Lipid bilayer walls form spontaneously when lipid molecules are dispersed in water, which allows both polar and nonpolar molecules to be enclosed in the polar interior of the liposome or the nonpolar core of the lipid membrane^4^. The unique structure of liposomes allows the delivery of active compounds to target sites much more rapidly and in higher doses than do traditional preparations. They also protect these compounds more effectively from thermal and oxidative degradation, light, pH changes, and enzymes^5^. Each type of encapsulated molecule has its own encapsulation mechanism, which must be understood in order to prepare effective liposomes. PEGylated liposomes have many advantages over conventional liposomes, having longer action in the body, better accumulation in cells, reduced toxicity to healthy tissues, and increased stability^6–8^. However, they also have disadvantages, such as reduced cellular uptake, the potential to cause pseudoallergies, and uneven distribution in tissues^9–11^ These challenges show the need for further research and optimization in order to improve the safety and effectiveness of PEGylated liposomes. Phytosterols, also known as plant sterols, are bioactive compounds found in vegetable oils, fats, nuts, and some vegetables. Their chemical structure is similar to that of cholesterol, allowing them to contribute to lowering cholesterol levels in the blood by blocking its absorption^12^. The recommended intake of plant sterols in the diet is about 250–400 mg/day, with significant variability^13^which is similar to typical levels of cholesterol consumption. Phytosterol concentrations in plasma are lower than those of cholesterol, mainly due to their limited intestinal absorption. Less than 5% of plant sterols and less than 0.5% of stanols are absorbed in the human digestive system, while the absorption rate for cholesterol is about 50–60%^*14*^ .In order to achieve a cholesterol-lowering effect, it is thus necessary to consume at least 2–3 g of phytosterols per day^15^. Unfortunately, plant sterols undergo autoxidative degradation, leading to the formation of phytosterol oxidation products (POPs), also known as oxyphytosterols^16^.These products form during the storage and production of food, and are absorbed into human plasma, where they can be detected^17^. Oxyphytosterols exhibit atherogenic and cytotoxic properties^18^and their proinflammatory effects and ability to induce oxidative stress have been confirmed^19–21^. To date, few in vivo studies on humans have been conducted to identify which encapsulation methods yield the best results for absorption effects in the digestive tract and which substances added to food are most stable during storage and processing. It has been confirmed that phytosterols, as well as their esters with fatty acids, can be encapsulated in conventional liposomes^22^. During tests of storage and frying, stigmasterol encapsulated in liposomes degraded, forming oxyphytosterols. Some of these degradation products have a negative impact on human health^23–25^. Food safety and health are crucial for consumers, so a safe delivery form for sterols must be developed to prevent thermal–oxidative degradation and to enhance their absorption in the digestive tract. The aim of this study was to examine PEGylated liposomes containing free stigmasterol and its esters with saturated fatty acids subjected to lyophilization and subsequent heating at temperatures simulating storage (60 °C) and frying (180 °C). Stigmasterol was chosen as a representative phytosterol on account of the presence of two double bonds in its chemical structure: one in the B ring and the other in the side chain. The chemical structure of stigmasterol affects its oxidative stability.

## Results and discussion

**Fig. 1:**
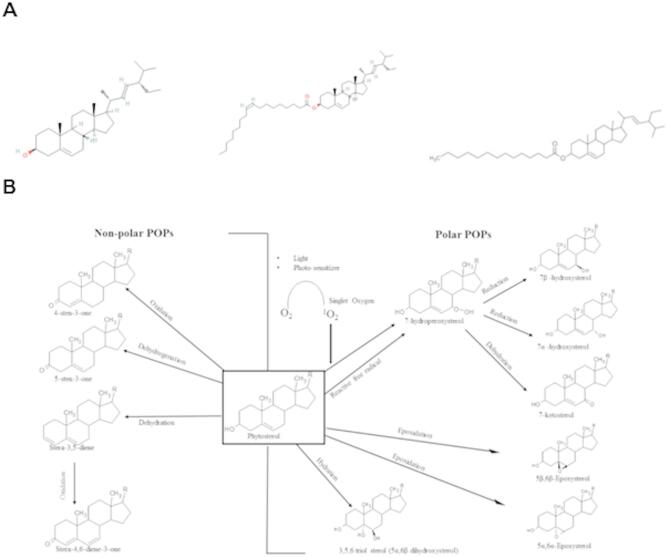
(**A**) Chemical structures of stigmasterol (ST) and its esters with myristic acid (ME) and oleic acid (OE), (**B**) formation of the main sterol oxidation products.

The chemical structures of free stigmasterol and its esters with myristic and oleic acids are presented in Fig. 1A. The PEGylated liposomes we have prepared are examples of plant sterol carriers that can enhance their thermo-oxidative stability. The general scheme of sterol oxidation is shown in Fig. 1B. Our previous studies have shown that the esterification of stigmasterol with a saturated acid inhibits the degradation of stigmasterol during its thermal oxidation^26,27^. Encapsulation of these compounds in PEGylated liposomes would be expected to significantly increase their stability.

### TEM/SEM observations

**Fig. 2:**
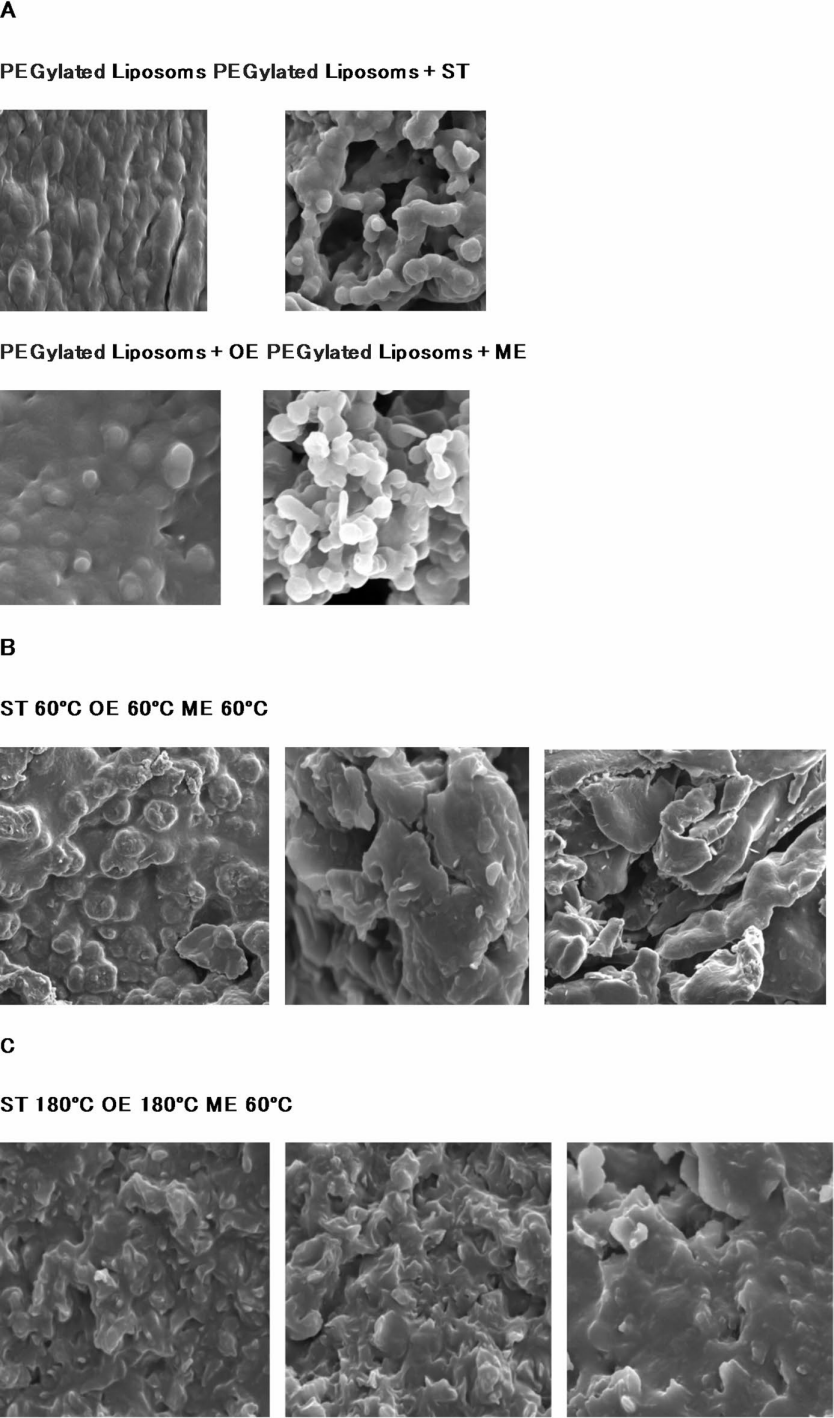
Microscope imagery of the PEGylated liposomes. (**A**) Transmission electron microscopy (TEM) of PEGylated liposomes encapsulated with stigmasterol (ST), myristate stigmasterol (ME), and oleate stigmasterol (OE), (**B**) scanning electron microscopy (SEM) of liposomes encapsulated with stigmasterol (ST), myristate stigmasterol (ME), and oleate stigmasterol (OE) after being maintained at 60 °C for eight hours, (C) SEM imagery of liposomes encapsulated with stigmasterol (ST), myristate stigmasterol (ME), and oleate stigmasterol (OE) after holding at 180 °C for eight hours.

TEM microscopy was used to determine the microstructures of the empty PEGylated liposomes prepared from dipalmitoylphosphatidylcholine (DPPC), PEGylated liposomes encapsulated with stigmasterol (ST), stigmasteryl myristate (ME), and stigmasteryl oleate (OE) before heating; these images are presented in Fig. 2A. These images show that the liposomes were successfully prepared. All the samples were nanosized, spherical, and possessed smooth surface. ST liposomes were the smallest, while the ME and OE liposomes were similar.

The theoretical phase transition temperature for DPPC has been determined to fall within the 40–44 °C range (Chen et al., 2013; Chen et al., 2018). SEM images of PEGylated liposomes containing ST, ME, and OE show the morphological changes they have undergone after being heated to 60 °C and 180 °C (Fig. 2B and C). The effect of elevated temperatures on these nanoparticles is of great importance for food products. As shown in Fig. 2B and C, PEGylated liposomes exposed to temperatures of 60 °C and 180 °C underwent significant morphological changes. DPPC likely degraded. The degree of degradation of the compounds encapsulated in the PEGylated liposomes has been described in this study.

### Zeta potential and hydrodynamic diameter of the pegylated liposomes

The zeta potential (ZP) of the PEGylated liposomes with stigmasterol (ST), stigmasteryl myristate (ME), and stigmasteryl oleate (OE) is presented in Table 1. In the PEGylated liposomes containing ST, a slight positive ZP (0.5 mV) is observed, as is typical of DPPC^22^.For the PEGylated liposomes with ME and OE, a reduction in ZP (to 0.3 mV and 0.4 mV, respectively) was seen, indicating a shift towards a negative charge. A similar trend was reported by Jovanović et al. (2018) when cholesterol and β-sitosterol were incorporated into DPPC membranes. In our experiment, this phenomenon may result from the stronger interactions of stigmasterol esters than free stigmasterol with the hydrophobic part of DPPC liposomes. This is a direct effect of their increased lipophilicity and the addition of PEG2000 to their structure; it may cause disruption of the DPPC gel phase and a consequent reorganization of the phospholipid groups. It should be noted that the relatively low positive zeta potential values of DPPC observed in the presence of stigmasterol and its esters do not favor liposome stability.

**Table 1:** Potential zeta and hydrodynamic diameters of pegylated liposomes loaded with stigmasterol (ST), stigmasterol myristate (ME), and stigmasterol oleate (OE) before and after holding them at 60 °C and 180 °C for eight hours.

Samples	Unheated	Held at 60 °C	Held at 180 °C
*Zeta potential (mV)*			
ST	0.5 ± 0.05	–28.6 ± 0.6	− 31.1 ± 0.5
ME	0.3 ± 0.01	− 23.8 ± 0.1	− 33.8 ± 1.5
OE	0.4 ± 0.07	− 11.6 ± 1	− 30.9 ± 0.8
*Hydrodynamic diameter (nm)*			
ST	102.4 ± 0.6	113.8 ± 1.8	970.8 ± 93.4
ME	302.2 ± 9.5	235 ± 21.5	146.2 ± 4.2
OE	164.8 ± 4.9	286.3± 5.5	303.8 ± 8.2

Pronounced changes in the zeta potential values of the PEGylated liposomes were observed upon heating to 60 °C (Table 1). In the presence of free stigmasterol, a strong change in zeta potential was noted (from 0.5 mV to -28.6 mV), which may be due to the membrane structure undergoing a change toward greater fluidity, triggered by the presence of oxidation products. ZP changes in the presence of ME were similar, ranging from 0.3 mV to − 23.7 mV. However, ZP changes were smaller in the presence of OE (0.4 mV and − 11.6 mV, respectively). This demonstrates the protective role of stigmasterol esters against structural changes in the membrane caused by peroxidation processes. A shift in the zeta potential toward negative values was observed whenever PEGylated liposomes were heated to 180 °C (− 31.11 mV, − 30.9 mV, and − 33.8 mV for ST, ME, and OE, respectively, Table 1). These results allow us to conclude that neither free nor esterified sterols were able to protect the membrane from significant structural changes under these drastic oxidation conditions. Another important parameter of liposomes is their hydrodynamic diameter (Table 1), which was significantly higher in the presence of stigmasterol esters than in the free form (302.2 nm for ME and 164.8 nm for OE compared to 102.4 nm for ST). It can be observed that sterols increase the size of PEGylated DPPC liposomes with a multilamellar structure. Rudzińska et al. (2025) claim that the average liposome diameter increases with free sterol content, but decreases with the incorporation of sterol esters into liposomes. They suggest that ester molecules may replace some phospholipid molecules in the membrane or form lipid rafts, leading to tighter lipid packing in liposomes. This could apply to natural phospholipids, where membrane packing is imperfect due to varying hydrocarbon chain lengths, degrees of saturation, and the presence of head groups^22^. In the case of tightly packed DPPC liposomes, it was noted that sterols increase the membrane size through interactions between lipid chains near phospholipid head groups, formation of inter-lipid spaces, and membrane expansion^22^ .In our experiment, such phenomena may also be responsible for the increase in liposome size, and this is confirmed by the significant increase in the hydrodynamic diameter in the presence of ME (302.3 nm), compared to in the presence of ST (102.4 nm); this results from increased membrane fluidity or the formation of interlipid spaces by bent myristic acid residues. Heating liposomes to 60 °C and 180 °C led to an increase in the hydrodynamic diameter for ST (up to 970.8 nm at 180 °C) and a decrease in size for ME (down to 146.2 nm at 180 °C). A significant decrease in the hydrodynamic diameter of liposomes containing ME was observed with increasing temperature — from 302.2 nm in the unheated state to 235.0 nm after heating to 60 °C, and then to 146.2 nm after heating to 180 °C. These changes indicate a structural reorganization of the liposomes under thermal conditions, likely caused by degradation of myristic acid residues, increased membrane fluidity, and a shift in the hydrophilic–lipophilic balance. The size reduction may also result from tighter lipid packing or partial collapse of multilamellar structures. Additionally, the presence of PEG2000 may have contributed to the stabilization of smaller liposomal forms at elevated temperatures. The decrease in ME liposome size after heating reflects their greater susceptibility to structural changes caused by oxidation and thermal degradation of lipid components. These changes differ between free and esterified sterols.

### Stability of ST, ME, and OE

#### Stigmasterol degradation

**Fig. 3:**
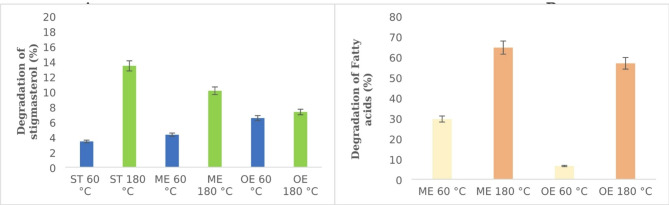
Degradation of (**A**) stigmasterol and (**B**) fatty acids after thermo-oxidation of PEGylated liposomes enriched with free stigmasterol (ST), myristic ester of stigmasterol (ME), and oleic ester of stigmasterol (OE) at 60 °C and 180 °C for eight hours.

PEGylated liposomes were encapsulated with three different molecules containing a plant sterol (stigmasterol). Stigmasterol was used on account of its chemical structure: it has two double bonds that affect its thermal oxidation. Additionally, the synthetic standard we used is in a pure form without other sterols. The changes in stigmasterol content in heated PEGylated liposomes depend on whether it is in a free or esterified form, the type of fatty acids used for esterifying the sterol, and the temperature. After heating the samples to 60 °C, stigmasterol degradation was found to be 3.4% in ST, 4.3% in ME, and 6.5% in OE (Fig. 3A). After heating to 180 °C, the lowest level of stigmasterol degradation (7.3%) was observed in OE, while the degradation levels in ST and ME was 13.4% and 10.1%, respectively (Fig. 3A). The sterol degradation level was lower for free sterols at lower temperatures, but higher at elevated temperatures. The degradation of stigmasterol in liposomes filled with stigmasterol oleinate was very similar at both temperatures. Oleic acid likely has a protective effect on stigmasterol at high temperatures. Similar results were presented by Kasprzak et al.^28^. Oleic acid also plays an important role in increasing the thermo-oxidative stability of stigmasterol in acylglycerols with stigmasterol residue^29,30^.

The level of degradation of stigmasterol during the storage of PEGylated liposomes (containing polyethylene glycol) at 60 °C was low. This suggests that the structure of the liposomal lipid with the addition of the PEG2000 bilayer may provide some protection against oxidation and degradation of sterols, such as stigmasterol. Liposomes, due to their lipid bilayer, can act as protective barriers, limiting the exposure of sterols to environmental factors that promote their oxidation. Such protective properties are especially important in the context of the development of functional foods, where the stability of active ingredients is crucial.

### Degradation of fatty acids

The level of degradation of myristic acid residue during heating was 29.5% and 64.5% at 60 °C and 180 °C, respectively—higher than for oleic acid residue (Fig. 3B), which decreased by 6.5% after heating to 60 °C and by 56.8% after heating to 180 °C. Despite the presence of a double bond in oleic acid, its loss was much less than that of myristic acid, indicating greater stability during heating. The results of studies conducted on PEGylated liposomes are consistent with previous studies on conventional liposomes. Liposomes containing oleic acid showed high resistance to lipid oxidation and were stable during long-term storage^22^. Our results show that the stability of fatty acids present in the phospholipids forming the liposome bilayer and those encapsulated in PEGylated liposomes differs: oleic acid, as part of the sterol ester in liposomes, exhibited better protective properties against stigmasterol degradation than did myristic acid.

### Stigmasterol oxidation products (SOP)

**Fig. 4:**
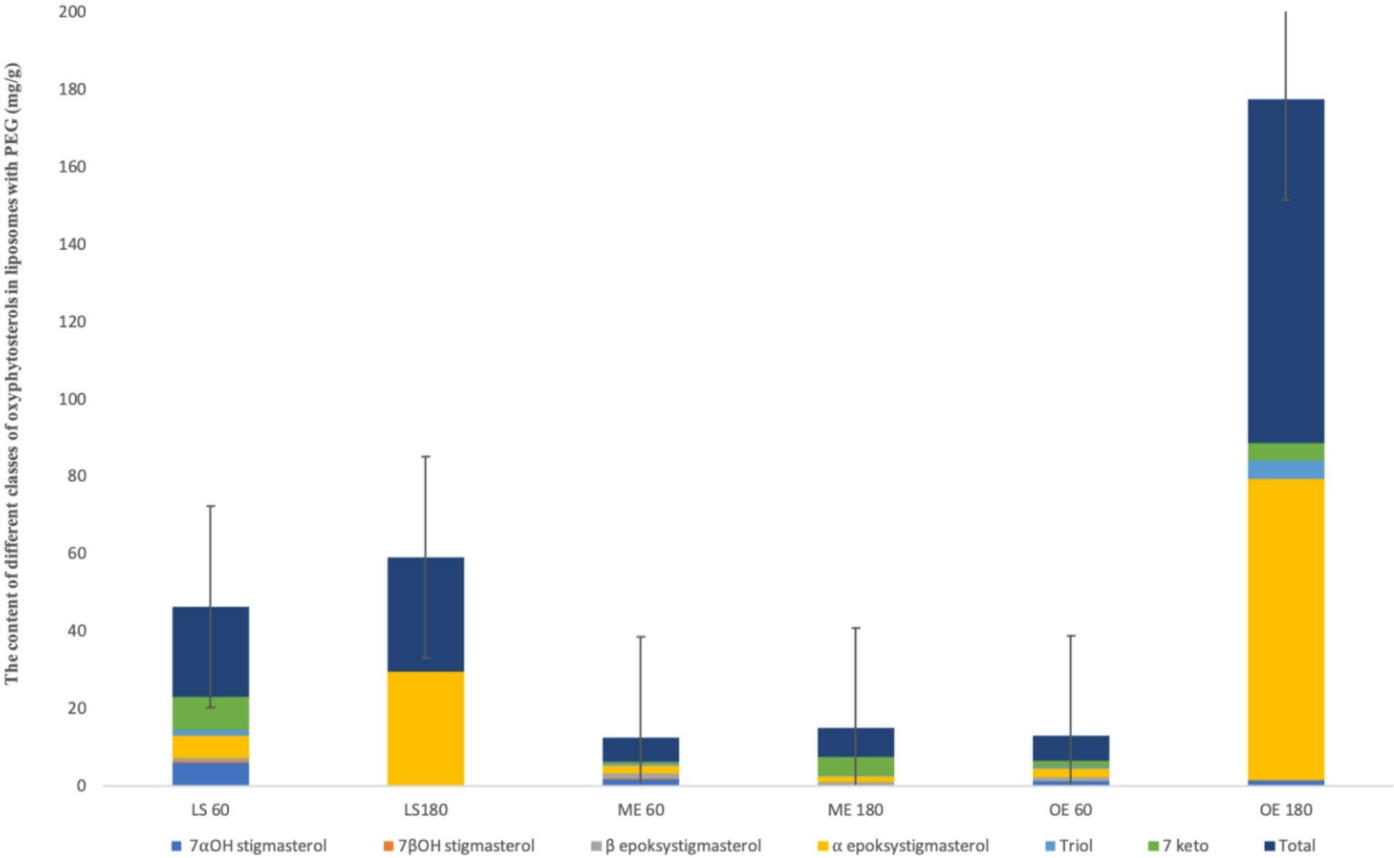
SOP formed during holding of PEGylated liposomes enriched with free stigmasterol (ST), myristic ester of stigmasterol (ME), and oleic ester of stigmasterol (OE) at 60 °C, and 180 °C for eight hours.

The SOP content of heated PEGylated liposomes encapsulated with free stigmasterol and its esters is presented in Fig. 4. SOPs were not detected in the liposomes before they were heated.

These results show that the SOP level in PEGylated liposomes heated to 60 °C ranged from 23.2 mg/g in liposomes containing free stigmasterol to 6.3 and 6.5 mg/g in liposomes containing stigmasterol myristate and oleinate, respectively. After heating to 180 °C, the total SOP content was significantly higher, ranging from 88.7 mg/g for OE to 7.5 and 29.6 mg/g for ME and ST, respectively. After heating standard stigmasterol to 60 °C for twelve hours and to 180 °C for eight hours, the total SOP content was found to be 1.1 mg/g and 2.4 mg/g, respectively^22^. The first stage of sterol autoxidation is the loss of a hydrogen atom at the allylic position C7 in the B-ring of the sterol structure, which can be removed by radicals. The addition of molecular oxygen leads to the formation of 7α- and 7β-peroxides. From these initial peroxides, a complex range of 7α- and 7β-hydroxysterols is then formed. Both isomers can be converted into 7-ketosterol. Another autoxidation pathway is through a reaction at the sterol’s Δ5,6 double bond, which leads to the formation of α- and β-epoxysterols as intermediate products, which are then converted into 3β,5α,6β-triol^31^. Six oxidation products of stigmasterol were identified in the heated PEGylated liposomes. The composition of SOP varied in dependence on the sample. Epoxy derivatives of stigmasterol heated to 60 °C were present in all types of PEGylated liposomes. While both isomers of 7-hydroxystigmasterol (7OHSt) had the highest levels for ST 60, all liposomes heated to 180 °C showed high levels of α-epoxySt. Esters also led to the formation of β-epoxySt. Holding PEGylated liposomes at 60 °C as an accelerated storage test, showed that liposomes with free stigmasterol were the most stable. The esterification of stigmasterol with myristic and oleic acids reduced their stability. Reverse results were obtained for PEGylated liposomes heated to 180 °C, where α-epoxystigmasterol and 7ketoSt predominated in ST. PEGylated liposomes with esters contained high levels of α-epoxySt, followed by β-epoxySt. The highest level of triolSt was detected in OE 180 heated to 180 °C, which was 4.7 mg/g, the highest of all the samples studied. There is limited information in the literature on the formation of oxidation products of phytosterols in liposomes containing plant sterols during thermal oxidation. Some important data was published in 2025 regarding the formation of oxidation products of stigmasterol and its esters during liposome formation, in which the above compounds were identified^22^.

### Principal component analysis (PCA)

Principal component analysis (PCA) was conducted to investigate the variability in the chemical composition of the samples depending on the heat treatment used (unheated, heated to 60°Cm and heated to 180 °C) and to identify the key variables responsible for these differences.

**Fig. 5:**
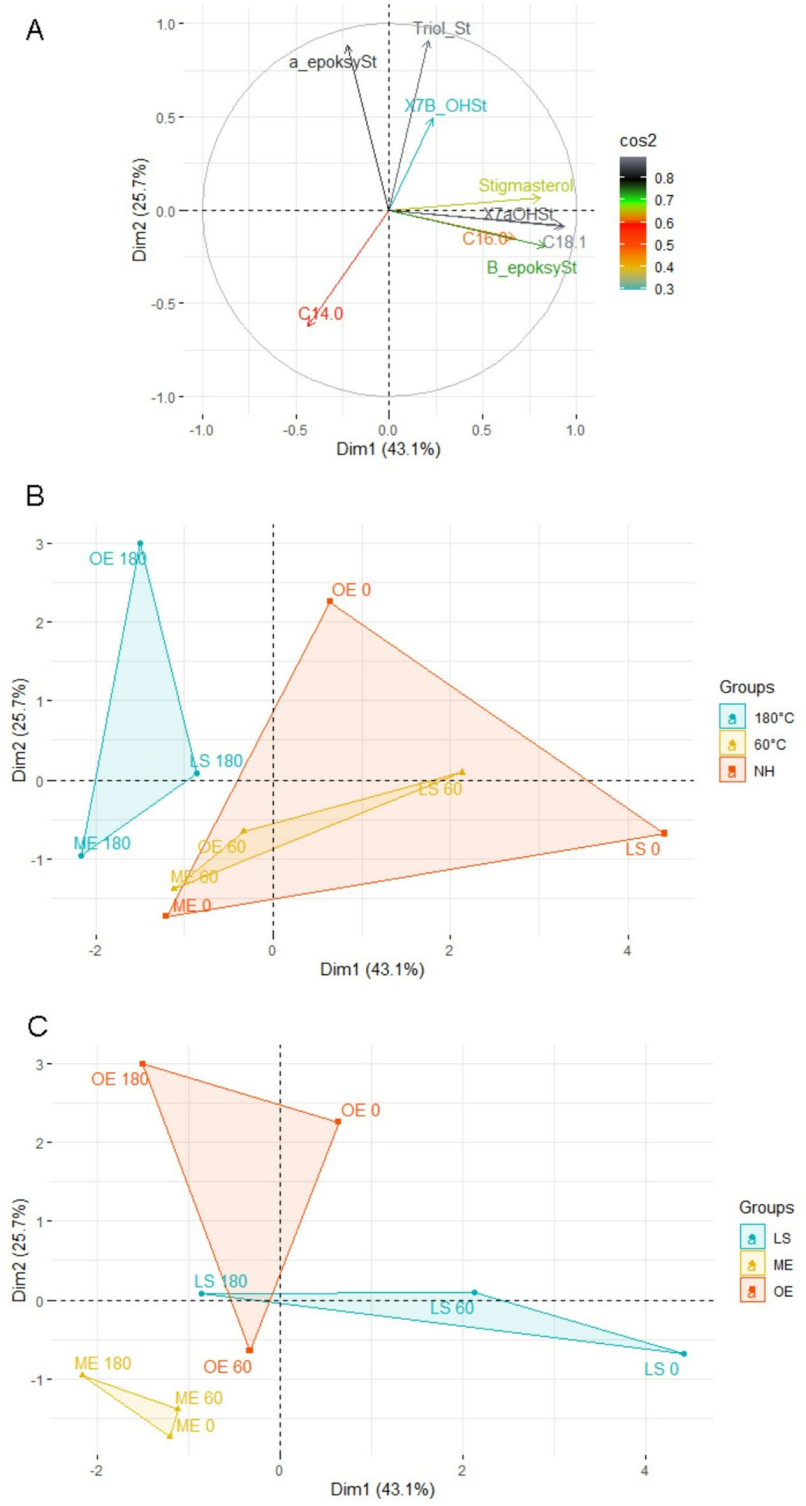
Principal component analysis (PCA) of the loading plot and score plot of the data from the fatty acids and phytosterol, along with the oxyphytosterol content of the unheated liposomes and of those heated at 60 °C and 180 °C. NH: unheated. LS (ST): liposomes encapsulated with free stigmasterol, ME: liposomes encapsulated with stigmasterol myristate, OE: liposomes encapsulated with stigmasterol oleate.

The PCA results show that the first two principal components explain of 68.8% of total variation (Dim1 = 43.1% of variance, Dim2 = 25.7% of variance). The first factor is mainly affected by variables associated with C18:1 fatty acid (0.94), 7α-OHSt (0.92) and stigmasterol (0.81). The second factor is driven by variables such as triolSt (0.91) and α-epoxySt (0.88). The variable PCA plot (Fig. 5A) highlights strong correlations between C16:0 and C18:1, as well as a negative correlation between these variables and C14.0, reflecting the compositional differences in chemical constituents. The group plot (Fig. 5B) reveals a clear separation between unheated (NH) samples and those treated at 60 °C and 180 °C. NH samples are more closely clustered, indicating the homogeneity of their chemical composition. The shift in sample positions along Dim1 (the x-axis) and Dim2 (the y-axis) with higher temperatures suggests significant changes in the chemical composition, particularly with respect to fatty acids (C16:0, C18:1) and sterols (stigmasterol, 7-α OH St). Figure 5C illustrates the relationship between groups of liposomes (LS (ST), ME, OE) and heat treatment temperatures, indicating varying impacts of thermal processing on the chemical composition, depending on the group. The OE group at 180 °C shows the largest deviation along the y-axis, suggesting a dominance of components such as stigmasterol triol and α-epoxy stigmasterol. In contrast, the ME group exhibits smaller compositional changes, indicating greater stability under a range of thermal conditions.

In summary, the PCA shows that the variability in the chemical composition of the samples is mainly driven by the temperature of the heat treatment, with fatty acids (C16:0, C18:1) and sterols (stigmasterol, 7α OH St) contributing the most to the differentiation among samples. These findings confirm the significant effect of temperature on the chemical properties of the samples.

## Conclusions

PEGylated liposomes encapsulating free stigmasterol, stigmasterol myristate, and stigmasterol oleate were prepared, and their stability was studied after heating to 60 °C and 180 °C to simulate storage and frying. TEM and SEM images showed significant changes in the morphological structure of the liposomes following lyophilization and thermal oxidation. ST liposomes were the smallest, while ME and OE liposomes were similar in size. The zeta potential and hydrodynamic diameter of the liposomes also underwent significant changes. We can conclude that the stigmasterol esters and the addition of PEG2000 protect against structural changes in the membranes on heating to 60 °C, but this was insufficient to protect the membranes after heating to 180 °C. The degradation of the compounds encapsulated in PEGylated liposomes depends on their structure. When the samples were heated to 60 °C, liposomes containing free stigmasterol were the least degraded, followed by liposomes containing stigmasterol myristate. However, at 180 °C, free stigmasterol degraded the most rapidly, while stigmasterol oleate was the most stable. Furthermore, the high level of oxyphytosterols in the samples heated to 180 °C could have led to negative effects in the liposomes. The results suggest that the use of PEGylated liposomes containing free phytosterols in foods that are not subjected to heat treatment is better than the use of esters. Food products subjected to frying will be least exposed to negative effects when phytosterol esters with oleic acid are used. It is likely that the addition of antioxidants to liposomes will improve the stability of phytosterols and reduce the formation of derivatives that adversely affect health.

## Materials and methods

### Materials

Stigmasterol (≥ 95%), myristic acid (≥ 98.0%), oleic acid (≥ 99%), all the solvents, sodium hydroxide, anhydrous pyridine, the catalysts dicyclohexylcarbodiimide (DCC) and 4-dimethylaminopyridine (DMAP), high-purity silica gel 70–230 mesh, PEG2000, standards of cholesteryl oleate (98%), 5α-cholestane, 19-hydroxy-cholesterol, and heptadecanoic acid methyl ester were purchased from Sigma-Aldrich (St. Louis, MO, USA). The internal standard 19-hydroxycholesterol was purchased from Steraloids (Newport, RI, USA). The silylation mixture of BSTFA [*N*, *O*-Bis(trimethylsilyl) trifluoroacetamide] with 1% TMCS (trimethylchlorosilane) was obtained from Fluka Chemie, while the SEP-PAK amino cartridges were sourced from Waters (Milford, USA). Dipalmitoylphosphatidylcholine (DPPC) was purchased from Avanti Polar Lipids (Birmingham, AL, USA).

### Esterification

Stigmasteryl esters were synthesized with myristic and oleic acids through esterification^26,32^. Briefly, stigmasterol was dissolved in dichloromethane, and then the catalysts (500 mg DCC and 15 mg DMAP) and fatty acids were added. The reaction was conducted under an argon atmosphere at room temperature for 24 h in the dark. Once the reaction completed, distilled water was added, and the mixture was subjected to extraction. The lower layer was collected, and the purification process was repeated three times. The solvent was then evaporated, and the remaining product was dissolved in hexane. The purified ester then underwent chromatography on a silica gel column, and the product’s purity was verified using TLC (comparing it with a cholesteryl ester standard). The quality of the esters produced was assessed using GC–MS^22,38^.

### Preparation of pegylated liposomes

PEGylated liposomes were prepared according to the method described by Cieślik-Boczula^39^ with a modification involving the addition of PEG2000 PE. Ten milliliters of organic solvent (chloroform: methanol in a 2:1, V: V ratio) were added to the mixture and the organic solvent was removed. One milliliter of buffer solution was added to the resultant lipid film at a temperature 10 °C above the gel–liquid–crystalline phase transition temperature. The final phospholipid concentration lay between 10 and 30 mg/mL in the phosphate buffer. Lipid dispersion was ultrasonicated using InterSonic water bath sonicator (Intersonic Sysytems, Poland) at 80 kHz for 30 min. at 55°C^22,39^.

### Freeze-drying of pegylated liposomes

The liposomes were dried using an Alpha 2–4 LD freeze-dryer (Christ, Germany) with a freeze temperature of –18 °C and a drying temperature of –20 °C for 20 h. Desiccation was then performed at 5 °C for 4 h^22^.

### Zeta potential and hydrodynamic diameter of the pegylated liposomes

Dynamic light scattering (DLS) is a widely used technique for measuring the diameter of particles exhibiting Brownian motion in a solution. To measure the hydrodynamic diameter of PEGylated liposomes and the zeta potential, a Zetasizer Nano ZS-90 (Malvern, UK) was used. The samples were diluted ten times with ultrapure water and placed in the spectrometer immediately after dilution. The data are expressed as means ± standard deviations^22^.

### Thermal stability

PEGylated liposomes were heated at temperatures of 60 °C and 180 °C to determine their thermal stability under simulated storage and frying conditions. The heating time was eight hours. After heating, the ampoules in which the samples were heated were sealed and cooled to room temperature. The experiment was conducted in two replicates^22,29^.

### Microscopy

Transmission electron microscopy (TEM) is one of the most commonly used methods for investigating the size and shape of liposomes. TEM imaging was performed using an FEI Tecnai G2 20 X-TWIN transmission electron microscope (Thermo Fisher Scientific) operating at an accelerating voltage of 200 kV. A 5 µL droplet of a 100× diluted liposome solution was placed on a 400-mesh carbon-coated microscope grid. Excess liquid was removed after one minute. The liposomes were then negatively stained for fifty seconds with a 2% solution of uranyl acetate dissolved in distilled water. The liposome samples were placed on tables with previously glued carbon discs for observation under a scanning electron microscope (SEM). The samples were then coated with gold using a Quorum Q 150R S sputtering machine. Observations and image registration were performed under high vacuum conditions in a Zeiss EVO 10 scanning electron microscope^22^.

### Sterols

Stigmasterol content was determined using AOCS Official Method Ch 6–91 (2009) and AOCS Official Method Cg 5–-97 (2009)^35,36^. The samples were saponified with 2 M methanolic KOH for 18 h. Phytosterol was then extracted using hexane/methyl tert-butyl ether (1:1, v/v). The extracts were evaporated, and the samples were silylated using BSTFA + 1% TMCS. A gas chromatograph (Agilent Technologies 7820 A) with a flame ionization detector was used for separation, equipped with a DB-35MS capillary column (25 m × 0.20 mm, 0.33 μm; J&W Scientific, Folsom, California, USA). A 0.5 µL sample was injected in splitless mode. The oven was initially programmed at 280 °C, held for 20 min, heated to 290 °C at a rate of 0.7 °C/min and held for 5 min, and then ramped up to 320 °C at a rate of 30 °C/min, where it was held for 5 min. The carrier gas was hydrogen (2 mL/min). Stigmasterol was identified by comparing relative retention times with those of standards derivatized by the same procedure as the samples. 5α-cholestane was used as a standard, and stigmasterol was then used for comparison during retention with the standards^22,25,29,30^.

### Fatty acids

The fatty acids profile was investigated using gas chromatography following the AOCS Official Method Ce 1k-07 (2007)^37^ and employing an Agilent Technologies 8890 gas chromatograph with a flame ionization detector (FID). The column was an SPTM-2560 capillary column (100 m × 0.25 mm × 0.2 μm; Supelco, Bellefonte, PA, USA). The carrier gas was hydrogen (1.5 mL/min). For each analysis, 0.5 µL of the sample was injected. The oven temperature started at 60 °C (where it was kept for 1 min), and was then increased at 6 °C/min to 220 °C. The inlet and detector were both at 240 °C. A 100 µg mixture of fatty acid methyl esters (FAME Mix on SP -2560) was added to the sample as an internal standard^22,26^.

### Stigmasterol oxidation products (SOP)

The method described by Rudzińska et al.^38,39^ was used to determine the derivatives of the oxidized phytosterols. The lipid fraction was extracted using the Folch method with the addition of 0.006% BHT and then transesterified using sodium methoxide. After extraction with chloroform, the sample was fractionated on SEP-PAK NH2 columns, silylated, and analyzed using a gas chromatograph (Agilent Technologies 7820 A) equipped with a DB-35MS capillary column (25 m × 0.20 mm × 0.33 μm; J&W Scientific). The temperature was programmed to increase from 50 °C to 270 °C at 25 °C/min, then from 270 °C to 290 °C at 1 °C/min, before being kept at 290 °C for 95 min. 19-hydroxy-cholesterol was used as an internal standard. The samples were identified by comparing their relative retention times with those of standards derivatized by the same procedure as the samples^22,28–30^.

## Statistical analysis

The experiments and analysis were performed in three independent replicates and the data presented here are mean values with standard deviations (± SD). The statistical analysis was performed using Statistica version 13.3 (Statsoft, Tulsa, OK, USA). The significance of the main effects was determined by one-way analysis of variance (ANOVA).

## Data Availability

The Data Collection of the Poznań University of Life Sciences in RepOD repository. https://repod.icm.edu.pl/dataset.xhtml? persistentId=doi:10.18150/W8CBNJ.
